# Predictive Nomogram for Early Recurrence after Pancreatectomy in Resectable Pancreatic Cancer: Risk Classification Using Preoperative Clinicopathologic Factors

**DOI:** 10.3390/cancers12010137

**Published:** 2020-01-06

**Authors:** Naru Kim, In Woong Han, Youngju Ryu, Dae Wook Hwang, Jin Seok Heo, Dong Wook Choi, Sang Hyun Shin

**Affiliations:** 1 Division of Hepatobiliary-Pancreatic Surgery, Department of Surgery, Samsung Medical Center, Sungkyunkwan University School of Medicine, Seoul 06351, Korea; naroo0107@naver.com (N.K.); cardioman76@gmail.com (I.W.H.); honeymoneykr@naver.com (Y.R.); jsheo.md@gmail.com (J.S.H.); dw7722.choi@samsung.com (D.W.C.); 2 Division of Hepatobiliary and Pancreatic surgery, Department of Surgery, Asan Medical Center, University of Ulsan College of Medicine, Seoul 05505, Korea; dwhwang@amc.seoul.kr

**Keywords:** pancreatic cancer, pancreatic ductal adenocarcinoma, recurrence, nomogram, neoadjuvant therapy

## Abstract

The survival of patients with pancreatic ductal adenocarcinoma (PDAC) is closely related to recurrence. It is necessary to classify the risk factors for early recurrence and to develop a tool for predicting the initial outcome after surgery. Among patients with resected resectable PDAC at Samsung Medical Center (Seoul, Korea) between January 2007 and December 2016, 631 patients were classified as the training set. Analyses identifying preoperative factors affecting early recurrence after surgery were performed. When the *p*-value estimated from univariable Cox’s proportional hazard regression analysis was <0.05, the variables were included in multivariable analysis and used for establishing the nomogram. The established nomogram predicted the probability of early recurrence within 12 months after surgery in resectable PDAC. One thousand bootstrap resamplings were used to validate the nomogram. The concordance index was 0.665 (95% confidence interval [CI], 0.637–0.695), and the incremental area under the curve was 0.655 (95% CI, 0.631–0.682). We developed a web-based calculator, and the nomogram is freely available at http://pdac.smchbp.org/. This is the first nomogram to predict early recurrence after surgery for resectable PDAC in the preoperative setting, providing a method to allow proceeding to treatment customized according to the risk of individual patients.

## 1. Introduction

Pancreatic ductal adenocarcinoma (PDAC) is one of the most lethal diseases worldwide, and is the fifth leading cause of cancer deaths in Korea [1]. Although surgical resection is an essential factor in providing a cure, only a minority of PDAC cases are diagnosed at a stage that can still benefit from surgical resection [2]. Further, even in patients considered eligible for surgical resection, early recurrence within 12 months after surgery has been reported to occur in 50% to 60% and the 5-year survival rate has been reported to be only 20% to 30% [3].

The resectability criteria for PDAC without distant metastasis were proposed by the National Comprehensive Cancer Network (NCCN), and tailored therapeutic strategies according to the classification are applied to improve the prognosis [4]. However, even in cases classified as resectable according to the NCCN guideline, high recurrence rate and low survival rate after surgery have been reported. For that reason, this current guideline recommends considering neoadjuvant therapy in high-risk patients of resectable disease. But, the high-risk features are presented with an ambiguous definition, such as very highly elevated CA19-9, large primary tumor, large regional lymph nodes, excessive weight loss, or extreme pain. The survival of patients with PDAC is closely related to recurrence, and early recurrence after surgery is one of the typical characteristics of PDAC. Therefore, in order to distinguish high-risk patients, it is necessary to classify the risk factors for early recurrence and to develop a tool for predicting the initial outcome after surgery beyond the criteria for determining the feasibility of surgical resection.

In the present study, we focused on the prediction of early recurrence, not survival, and we attempted to predict the postoperative outcome before the initiation of treatment. The purpose of this study was to develop a risk prediction model for early recurrence of PDAC using preoperative factors to clarify the high-risk features in patients with resectable disease.

## 2. Patients and Methods

### 2.1. Patients Database

Between January 2007 and December 2016, a total of 833 consecutive patients with PDAC underwent pancreatectomy with a curative intent at Samsung Medical Center (Seoul, Korea). Their electronic medical records were retrospectively reviewed from a prospectively maintained electronic database system (MDB^©^, Seoul, Korea). This study was approved by the Institutional Review Board of Samsung Medical Center (approval no. 2018-10-125). Because this study aimed to extract preoperative factors affecting early recurrence after surgery in resectable PDAC, we excluded patients with initially borderline resectable/unresectable cancers according to the NCCN guideline [4], as well as patients who were lost to follow-up. Of the 753 patients with resectable PDAC after exclusion, 631 had all considered preoperative factors without omission and their data were used as the training set. Our Institutional Review Board (IRB) waived the need for written informed consent from the participants.

Follow-up data were also obtained from the records, and the disease-free survival (DFS) was measured from the time of surgery until the detection of a recurrence. For postoperative surveillance, contrast-enhanced abdominoperineal computed tomography (CT) and carbohydrate antigen 19-9 (CA19-9) levels were examined every 3 months during the first 2 years postoperatively and every 6 months thereafter at our institute. The diagnosis of recurrence was based on progressive soft tissue growth at specific sites and elevated CA19-9 levels [5]. When lesions of potential recurrent disease were detected, ^18^F-fluorodeoxyglucose positron emission tomography, chest CT, and/or biopsy were performed to confirm the diagnosis of recurrence. Because recurrent lesions of PDAC often have located in inaccessible area, most of recurrences were diagnosed by imaging. However, biopsy was performed in some patients who were not accurately diagnosed by imaging. Although a clear definition of the term ‘early recurrence’ is currently lacking, a previous study concluded that a recurrence-free interval of 12 months is the optimal threshold for differentiating between early and late recurrence [6]. Accordingly, early recurrence was defined as recurrence within the first year after surgery in the present study.

### 2.2. Preoperative Data

Tumor markers including carcinoembryonic antigen (CEA) level, CA19-9 level, white blood cell count, and platelet count were collected using the measurements that were closest to the operation and within at least 1 month before the surgery. Inflammation-based prognostic scores, including neutrophil-lymphocyte ratio (NLR) and platelet-lymphocyte ratio (PLR), were calculated [7]. NLR and PLR were divided into two groups based on the 75% quantile. Tumor size, tumor location, and abutment degree to the portal vein (PV)-superior mesenteric vein (SMV) were measured using preoperative CT scans.

### 2.3. Data Analysis and Statistical Methods

The chi-square test was used for categorical variables and Student’s t-test or the Mann-Whitney U-test was used for continuous variables. Disease-specific survival (DSS) and DFS were depicted using Kaplan–Meier curves. Cox proportional hazard regression analysis was used for estimating the effect of preoperative risk factors for early recurrence. A *p*-value of <0.05 was considered statistically significant. The performance of survival models for early recurrence was evaluated using the concordance index (c-index), the integrated area under the curve (iAUC), and a calibration plot using the bootstrap samples from the training set. Further, a nomogram-based prediction of DFS was developed. First, preoperative risk factors for early recurrence were selected from the training set. If the *p*-value was <0.05 in a univariable analysis, variables were included in multivariable analysis and in the nomogram. Second, calibration curves with a thousand bootstrap replications were plotted, as well as for the observed empirical versus the predicted probability. Finally, based on the optimal cut-off value obtained from the Youden index, the positive and negative likelihood ratios were calculated, which led to the development of a Fagan’s nomogram for estimating the posttest probability of a patient to have a disease [8]. Recurrence analysis was executed using SAS version 9.4 (SAS Institute Inc., Cary, NC, USA). The nomogram was established based on the results of multivariable Cox’s regression analysis using R 3.5.1 (Vienna, Austria; http://www.R-project.org/).

## 3. Results

### 3.1. Demographic Features of Preoperative Factors

The clinicopathological characteristics of the overall patients (*n* = 753) and the training set (*n* = 631) are shown in Table 1. Early recurrence was identified in 394 (52.3%) of overall 753 patients, 45 (11.4%) of whom were diagnosed by biopsy. In the overall patients, the CA 19-9 level and NLR were significantly higher (*p* < 0.001 and *p* = 0.016, respectively), and the measured tumor size on CT was larger (*p* < 0.001) in the early recurrence group. The tumor differentiation was also significantly different (*p* < 0.001) between the two groups. In the training set (*n* = 631), the four factors mentioned above still showed statistically significant differences. Additionally, the PLR also showed a significant difference (*p* = 0.011).

### 3.2. Survival Analysis

**Figure 1:**
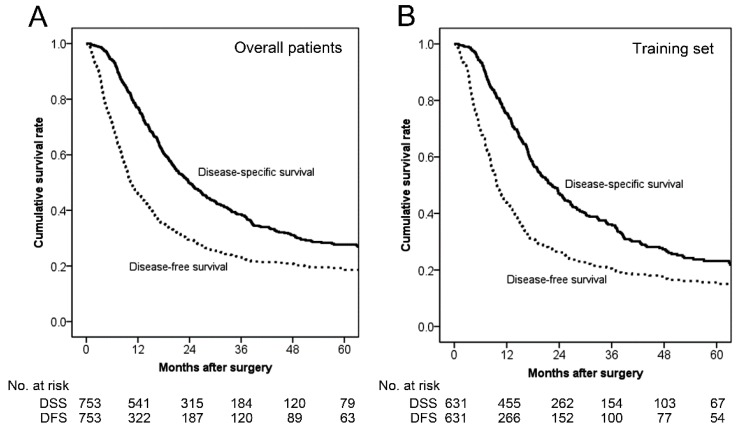
Kaplan-Meier survival curves of the overall patients and the training set. (**A**) In the overall patients (*n* = 753), the median disease-specific survival (DSS) was 23.8 months, and the 1-, 2-, and 5-year DSS rates were 77.0%, 49.9%, and 27.7%, respectively. The median disease-free survival (DFS) was 10.4 months, and the 1-, 2-, and 5-year DFS rates were 46.0%, 29.5%, and 18.9%, respectively. (**B**) In the training set (*n* = 631), the median DSS was 23.6 months, and the 1-, 2-, and 5-year DSS rates were 77.2%, 48.7%, and 26.0%, respectively. The median DFS was 10.4 months, and the 1-, 2-, and 5-year DFS rates were 45.6%, 28.6%, and 18.7%, respectively.

The median DSS in the overall patients and in the training set were 23.8 and 23.6 months, respectively (Figure 1). The median DFS and 1-year DFS rate in the overall patients were 10.4 months and 46.0%, respectively. In the training set, the median DFS and 1-year DFS rate were 10.4 months and 45.6%, respectively.

### 3.3. Preoperative Risk Factors and Establishment of a Nomogram 

**Figure 2:**
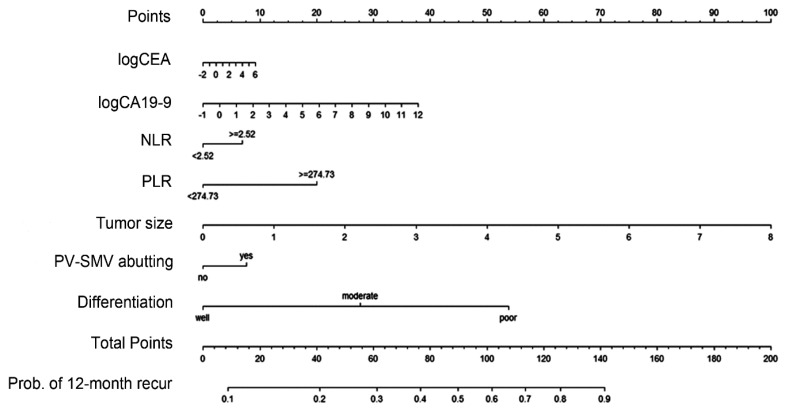
Nomogram for predicting early recurrence (within 12 months) after pancreatectomy using preoperative parameters in resectable pancreatic ductal adenocarcinoma. CEA: carcinoembryonic antigen; CA: carbohydrate antigen; NLR: neutrophil-lymphocyte ratio; PLR: platelet-lymphocyte ratio; PV-SMV: portal vein-superior mesenteric vein

**Figure 3:**
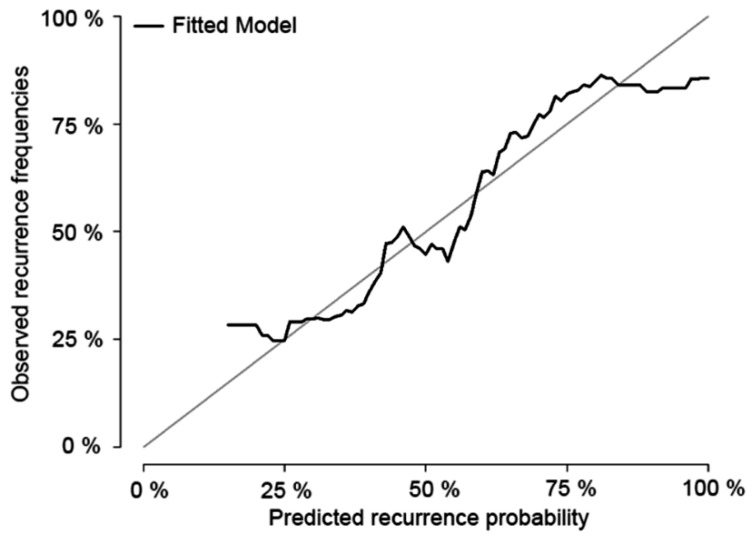
Calibration plot of the nomogram. One thousand bootstrap resamplings were used to validate the established nomogram. The concordance index (c-index) was 0.665 (95% confidence interval [CI], 0.637–0.695), and the incremental area under curve was 0.655 (95% CI, 0.631–0.682).

Analyses identifying the preoperative factors affecting early recurrence after surgery were performed in the training set (Table 2). When the *p*-value estimated from univariable Cox’s proportional hazard regression analysis was <0.05, the variables were included in multivariable analysis and used for establishing a nomogram, including logCEA, logCA19-9, NLR, PLR, tumor size on CT, PV-SMV abutment, and tumor differentiation. Considering the hazard ratio estimated from multivariable analysis for each factor, the total points were summed and the early recurrence probabilities were calculated based on these points. The nomogram based on the Cox model is shown in Figure 2. The nomogram predicted the probability that a patient will have a recurrence within 12 months after surgery for resectable PDAC. One thousand bootstrap resamplings were used to validate the established nomogram (Figure 3). The c-index was 0.665 (95% confidence interval [CI], 0.637–0.695), and the iAUC was 0.655 (95% CI, 0.631–0.682). We developed a web-based calculator, and the nomogram is freely available at http://pdac.smchbp.org/.

### 3.4. Postoperative Outcomes

The postoperative outcomes are described in Table 3. The early recurrence group had significantly more advanced T and N stages than the no or late recurrence group (all *p* < 0.001). However, there were no significant differences between the two groups in factors that could affect the prognosis or the results of this study, such as resection margin status or adjuvant therapy (*p* = 0.246 and *p* = 0.338, respectively). Postoperative complications, which were graded by Clavien–Dindo complication classification [9,10], were not also significantly different between two groups. In terms of recurrence patterns, there were more systemic recurrences in the early recurrence group (*p* < 0.001).

## 4. Discussion

Patients with resected PDAC are very likely to die of their disease because PDAC is notorious for aggressive invasion, early metastasis, and subsequent poor clinical outcomes. To date, many researchers have studied the factors that can predict the prognosis of PDAC and have made efforts to improve patient survival. However, the studies have shown inconsistent results depending on the research institute and the study cohort, and the predictors have not been able to directly contribute to improving prognosis because most of them are unmodifiable factors [3,11,12]. To complement the inconsistency of the prognostic factors of PDAC and to more accurately predict the survival possibility, predictive nomograms developed by analysing the contribution of each factor have been reported [13,14,15,16]. Although these outcomes may contribute to predicting the overall outcomes of a patient, there are still limitations in contributing to prognosis improvement. Therefore, we focused on the prediction of early recurrence, not survival. To transform prognostic factors with inconsistencies and unmodifiable characteristics into a tool that can be applied clinically, we developed a risk prediction nomogram for early recurrence of resectable PDAC in the preoperative setting (Figure 2).

As neoadjuvant therapy (NAT) for PDAC progresses, its role is also evolving. Previous studies have shown that NAT downstages some cases of initially borderline resectable or even locally advanced PDAC to a point wherein they become eligible for surgery [17,18,19,20]. Furthermore, some centres have taken the view that nearly all patients who appear to have potentially resectable PDAC should be considered for NAT on account of the inaccuracy of imaging, high rates of positive margins, and poor survival [21]. A recent meta-analysis showed that NAT for resectable PDAC seemed to improve overall survival in intention-to-treat and per-protocol analyses; however, the study showed that the overall resection rate was significantly lower with NAT than with upfront surgery (66.0 versus 81.3%, *p* < 0.001) [22]. Although the meta-analysis involved many considerations because it included both resectable and borderline resectable PDAC cases and most of the studies utilized older chemotherapy regimens, the low resection rate should be particularly carefully considered in patients with initially resectable PDAC. Low resection rates may be a basis for avoiding unnecessary surgery in some patients; however, they might lead to a loss of treatment opportunity in others. Therefore, we believe that it is timely to consider criteria that will be the clear boundary for NAT conducted in the setting of resectable PDAC.

**Figure 4:**
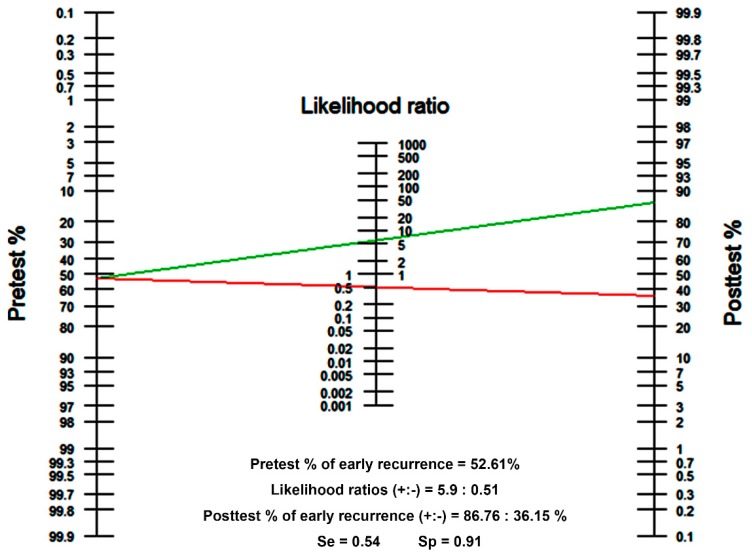
Fagan’s nomogram. The pretest probability of early recurrence is 52.61%. When the cut-off value of the predicted probability of early recurrence (within 12 months) is set to 0.71, the likelihood ratios of positive and negative test results are 5.9 and 0.51, respectively. The positive posttest probability of early recurrence (green line) is 86.76%, and the negative posttest probability of early recurrence (red line) is 36.15%.

In the present study, we devised a tool for predicting posttreatment outcomes before starting treatment, which can be used to determine the treatment direction. To optimize the predictive value of the designed nomogram, we determined the cut-off value using the Youden index. When the cut-off value of the nomogram-predicted probability of early recurrence (within 12 months) was set to 0.71 (Figure 4), the estimated diagnostic sensitivity and specificity were 54% and 91%, respectively. The likelihood ratios of positive and negative test results were calculated as 5.9 and 0.51, respectively. If a patient with an estimated early recurrence rate (pretest probability) of 52.61% tests positive, the posttest probability that the patient truly has early recurrence would be approximately 86.76% (green line). Alternatively, if the patient tests negative, the posttest probability would be approximately 36.15% (red line). In this way, it is expected that the nomogram can be used not only for prediction but also as a tool for determining the direction of actual treatment and customized treatment.

For example, an endoscopic ultrasound-guided biopsy-proven poorly differentiated 3.3-cm lesion in the pancreatic head with SMV abutment, white blood cell count of 5400/μL (neutrophil count of 69% and lymphocyte count of 19.4%), platelet count of 250/μL, CEA level of 11.2 ng/mL, and CA19-9 level of 486 U/mL would yield a total of 136 points and the estimated early recurrence probability would be 84.4% (Figure 2). The estimated probability of 84.4% exceeds the cut-off value of 0.71, and this patient may be classified as high-risk of early recurrence. We could then recommend neoadjuvant therapy to this high-risk patient with resectable PDAC.

This study has several limitations. First, measurement errors may have occurred, because there might be inaccuracies in measuring the tumor size on CT scans. Although the magnitude of the error does not always match, we used preoperative values in the present study and confirmed that this approach had sufficient prognostic value. Likewise, because we used only preoperative factors, postoperative factors that could affect the prognosis were not considered. However, as shown in Table 3, the resection margin and adjuvant therapy, which may artificially affect the results, did not differ between the two groups. Second, there was a possibility of selection bias because this study was conducted in a training set with only patients who did not have omitted data. However, most of the factors did not show any differences, and the present study did not aim to investigate the characteristics of consecutive patients but to investigate the characteristics of individual patients according to the recurrence classification. Third, although the nomogram was devised using a large number of patients at a large tertiary centre and validation using bootstrapping was attempted, it was not possible to obtain enough patient numbers to perform sufficient validation. Verification will be necessary through validation, and it is worthwhile to test newly developed tools through deep learning using artificial intelligence beyond the statistical limits. In addition, in order to overcome the limitations of retrospective study with the characteristics of selection bias and incomplete data, we think that further research on prospective validation and controlled study, as well as external validation, will be needed.

## 5. Conclusions

In the present study, we attempted to develop a nomogram for predicting early recurrence after surgery using only preoperative clinicopathologic data. For now, this nomogram is meaningful as a tool that can be more widely applied because it uses easily accessible data. This tool is expected to be able to identify patients who are classified as morphologically resectable but have high-risk features of early recurrence, and it can provide a method to allow proceeding to treatment customized according to the risk of individual patients, by predicting early recurrence before surgery rather than simply predicting prognosis. Further, more advanced forms of nomograms using more specific and advanced data, such as biomarkers, as well as the development of tools using deep learning with artificial intelligence are expected in the future.

## Figures and Tables

**Table 1:** Preoperative demographic features of the overall patients and the training set.

Factors	Overall Patients (*n* = 753)			Training Set (*n* = 631)		
	No or Late Recurrence (*n* = 359)	Early Recurrence (*n* = 394)	*p*	No or Late Recurrence (*n* = 299)	Early Recurrence (*n* = 332)	*p*
Age, years			0.534			0.612
Mean (±SD)	62.7 (±10.3)	63.1 (±10.2)		62.8 (±10.3)	63.2 (±10.1)	
Sex, n (%)			0.589			0.667
Male	209 (58.2)	237 (60.2)		176 (58.8)	201 (60.6)	
Female	150 (41.8)	150 (41.8)		123 (41.2)	131 (39.4)	
BMI, kg/m^2^			0.346			0.507
Mean (±SD)	23.1 (±3.0)	22.9 (±3.1)		23.0 (±3.0)	22.9 (±3.1)	
Underlying DM, n (%)			0.381			0.639
No	239 (62.9)	248 (62.9)		199 (66.5)	209 (63.0)	
Within 1 year	43 (12.0)	54 (13.7)		40 (13.4)	49 (14.7)	
Beyond 1 year	77 (21.4)	92 (23.4)		60 (20.1)	74 (22.3)	
CEA, n (%)			0.087			0.141
Normal (<5.0 ng/m)	281 (78.8)	293 (74.4)		269 (90.0)	286 (86.1)	
Elevated (≥5.0 ng/m)	31 (11.6)	49 (12.4)		30 (10.0)	46 (13.9)	
NA	47 (7.6)	52 (13.2)				
CA19-9			<0.001			0.004
Normal (<37 U/mL)	133 (37.0)	101 (25.6)		106 (35.4)	83 (25.0)	
Elevated (≥37 U/mL)	219 (61.0)	290 (73.6)		193 (64.6)	249 (75.0)	
NA	7 (2.0)	3 (0.8)				
NLR, n (%) ^†^			0.016			0.014
<2.52	281 (78.3)	278 (70.6)		224 (74.9)	219 (66.0)	
≥2.52	78 (21.7)	116 (29.4)		75 (25.1)	113 (34.0)	
PLR, n (%) ^†^			0.073			0.011
<274.73	280 (78.0)	285 (72.3)		287 (96.9)	302 (91.0)	
≥274.73	79 (22.0)	109 (27.7)		12 (4.0)	30 (9.0)	
PV-SMV abutment, n (%)			0.102			0.075
No	271 (75.5)	276 (70.1)		227 (76.9)	231 (69.6)	
Yes	88 (24.5)	118 (29.9)		72 (24.1)	101 (30.4)	
Tumour size on CT, cm			<0.001			<0.001
Mean (±SD)	2.47 (±1.03)	2.98 (±1.04)		2.41 (±0.93)	2.97 (1.05)	
Tumour location			0.602			0.272
Head	255 (71.0)	273 (69.3)		219 (73.2)	230 (69.3)	
Body or tail	104 (29.0)	121 (30.7)		80 (26.8)	102 (30.7)	
Differentiation			<0.001			<0.001
Well	38 (10.6)	16 (4.0)		34 (11.4)	15 (4.5)	
Moderate	239 (66.6)	232 (58.9)		205 (68.5)	196 (59.0)	
Poor or undifferentiated	70 (19.5)	135 (34.3)		60 (20.1)	121 (36.5)	
NA	12 (3.3)	11 (2.8)				

SD = standard deviation; BMI = body mass index; DM = diabetes mellitus; CEA = carcinoembryonic antigen; CA19-9 = carbohydrate antigen 19-9; NA = not available; NLR = neutrophil-lymphocyte ratio; PLR = platelet-lymphocyte ratio; PV = portal vein; SMV = superior mesenteric vein; CT = computed tomography; ^†^ NLR and PLR are divided into two groups based on the 75% quantile.

**Table 2:** Preoperative risk factor analysis in the training set (*n* = 631).

Factors	Univariable			Multivariable		
	*p*	HR	95% CI	*p*	HR	95% CI
Age, year	0.23	1.003	0.93–1.014			
Sex						
Male	Reference					
Female	0.68	0.955	0.766–1.190			
BMI, kg/m^2^	0.60	0.990	0.956–1.027			
DM						
No	Reference					
within 1 year	0.48	1.119	0.820–1.528			
beyond 1 year	0.44	1.110	0.851–1.447			
logCEA	0.04	1.150	1.008–1.312	0.69	1.027	0.897–1.177
logCA19-9	<0.001	1.113	1.056–1.174	0.015	1.039	0.975–1.107
NLR ^†^						
<2.52	Reference			Reference		
≥2.52	0.019	1.313	1.046–1.648	0.20	1.175	0.918–1.503
PLR ^†^						
<274.73	Reference			Reference		
≥274.73	0.004	1.748	1.200–2.545	0.028	1.590	1.501-2.405
Tumor size in CT, cm	<0.001	1.351	1.240–1.472	<0.001	1.337	1.222-1.463
PV-SMV abutment						
No	Reference			Reference		
Yes	0.047	1.268	1.003–1.602	0.140	1.195	0.943–1.514
Tumor location						
Head	Reference					
Body or Tail	0.32	1.125	0.891–1.420			
Differentiation						
Well	Reference			Reference		
Moderate	0.020	1.867	1.104–3.156	0.017	1.904	1.123–3.228
Poor or undifferentiated	<0.001	3.399	1.986–5.817	<0.001	3.490	2.032–5.995

HR = hazard ratio; CI = confidence interval; BMI = body mass index; DM = diabetes mellitus; logCEA = logarithm of carcinoembryonic antigen level; logCA19-9 = logarithm of carbohydrate antigen level; NLR = neutrophil-lymphocyte ratio; PLR = platelet-lymphocyte ratio; CT = computed tomography; PV = portal vein; SMV = superior mesenteric vein; ^†^ NLR and PLR are divided into two groups based on the 75% quantile.

**Table 3:** Comparison of postoperative factors between recurrence groups in the training set (*n* = 631).

Factors	No or Late Recurrence (*n* = 299)	Early Recurrence (*n* = 332)	*p*
T stage, n (%)			<0.001
T1	90(30.1)	46(13.9)	
T2	185(61.9)	215(64.8)	
T3	24(8.0)	70(21.1)	
T4	0(0.0)	1(0.3)	
N stage, n (%)			<0.001
N0	130(43.5)	93(28.0)	
N1	129(43.1)	135(40.7)	
N2	40(13.4)	104(31.3)	
Postoperative complications *, n (%)			0.101
No or Grade I	175(58.5)	216(65.1)	
Grade II or above	124(41.5)	116(34.9)	
Resection margin, n (%)			0.246
R0	232(77.6)	270(81.3)	
R1	67(22.4)	62(18.7)	
Adjuvant therapy, n (%)			0.338
No	97(32.4)	126(37.9)	
Yes	200(66.9)	205(61.8)	
NA	2(0.7)	1(0.3)	
Recurrence pattern, n (%)			<0.001
No	151		
Locoregional ^†^	53(36.1)	68(20.4)	
Systemic ^‡^	94(63.9)	265(79.6)	

NA = not available; * Postoperative complications were graded by Clavien–Dindo complication classification [9,10].; ^†^ Recurrence in the remnant pancreas or soft tissue around the pancreaticojejunostomy site, such as along the celiac or superior mesenteric artery.; ^‡^ Including single distant metastasis, multiple metastasis, and peritoneal seeding.
